# From silence to symphony: transcriptional repression and recovery in response to DNA damage

**DOI:** 10.1080/21541264.2024.2406717

**Published:** 2024-10-01

**Authors:** Kamal Ajit, Monika Gullerova

**Affiliations:** Sir William Dunn School of Pathology, University of Oxford, Oxford, UK

**Keywords:** DNA damage, transcription, RNA polymerase II, repression, re-activation

## Abstract

Genotoxic stress resulting from DNA damage is resolved through a signaling cascade known as the DNA Damage Response (DDR). The repair of damaged DNA is essential for cell survival, often requiring the DDR to attenuate other cellular processes such as the cell cycle, DNA replication, and transcription of genes not involved in DDR. The complex relationship between DDR and transcription has only recently been investigated. Transcription can facilitate the DDR in response to double-strand breaks (DSBs) and stimulate nucleotide excision repair (NER). However, transcription may need to be reduced to prevent potential interference with the repair machinery. In this review, we discuss various mechanisms that regulate transcription repression in response to different types of DNA damage, categorizing them by their range and duration of effect. Finally, we explore various models of transcription recovery following DNA damage-induced repression.

## Introduction

DNA is continuously damaged, either endogenously through replication errors, replication-transcription collisions, or reactive metabolites like reactive oxygen and nitrogen species, or exogenously by environmental mutagens such as ultraviolet (UV) light, X-rays, viral infections, and certain cancer drugs and chemicals. DNA damage can be classified as follows: single base changes, such as the deamination of cytosine to uracil, or depurination, which can alter the DNA sequence; structural distortions, such as intra- or inter-strand crosslinks; bulky lesions, which physically impede replication or transcription; and DNA strand breaks. Single-strand breaks (SSBs) are the most common form of DNA damage (occurring ~55,000 times per human cell per day), while double-strand breaks (DSBs) are among the most severe, potentially resulting in extensive DNA loss if left unrepaired [1].

UV-induced DNA damage leads to bulky lesions, which are repaired via the nucleotide excision repair (NER) pathway. NER operates through a series of well-coordinated steps, involving numerous proteins and enzymes. The process begins with damage recognition, through two sub-pathways. Global Genomic NER (GG-NER) repairs bulky lesions across the genome, with detection aided by the XPC complex, comprised of either RAD23A or RAD23B, together with CETN2. On the other hand, Transcription-Coupled NER (TC-NER) targets only lesions that block transcription, using the stalled RNA polymerase II and to signal recruitment of Cockayne syndrome proteins CSA and CSB. After damage recognition, the transcription factor II H (TFIIH) complex is recruited to bulky lesions. Its helicase activity locally unwinds the DNA around the lesion. Next, two endonucleases, XPF-ERCC1 and XPG, make incisions on the damaged strand, excising a 24–32 nucleotide-long fragment. DNA polymerases δ or ε, with auxiliary factors PCNA and RPA, fill the resulting gap, synthesizing the new DNA strand. Finally, DNA ligase seals the new strand, restoring the DNA to its undamaged state [2,3].

DSBs are detected by Ataxia Telangiectasia mutated (ATM) kinase, which triggers the onset of the DDR. The DDR causes cell cycle arrest by activating checkpoint proteins (CHK2) as well as p53-dependent transcription of cell cycle regulators and DNA repair proteins [1]. DSBs are mainly repaired by non-homologous end-joining (NHEJ) or homologous recombination (HR) pathways [4]. NHEJ is an error-prone repair mechanism active throughout the cell cycle [5,6]. NHEJ begins with the rapid binding of the Ku70-Ku80 proteins to the DNA ends, inhibiting end resection and triggering the recruitment of DNA-PKcs kinase, which tethers the ends together. The subsequent recruitment of downstream factors, such as Artemis, XRCC4, DNA ligase 4,then enables either direct ligation of the blunt ends or end trimming followed by ligation [7–10, 11]. In contrast, HR is a error-free mechanism active in the S and G2 phases, utilizing the sister chromatid as a template for repair. HR is initiated by the MRE11-RAD50-NBS1 (MRN) complex and its accessory factor CtIP. End resection is facilitated by various pretins including MRN, CtIP, BRCA1, and EXO1/DNA2 to generate single-stranded DNA (ssDNA) overhangs. These overhangs are protected by RPA until BRCA2 mediates RPA displacement by RAD51 to form a nucleoprotein filament, which invades the template DNA, generating a D-loop intermediate and faithfully restoring the damaged strand [6,8–10,12, 11].

Active transcription promotes DSB repair by facilitating a more accessible chromatin state and recruiting repair factors to the site of damage. This process is further enhanced by DNA damage-induced long non-coding RNAs (dilncRNAs) and damage-induced antisense RNAs (DARTs) [13–16,73]. During transcription, RNA polymerase II (RNAPII) opens the chromatin, making it more accessible for repair proteins. When RNAPII encounters a bulky lesion or DSB, it stalls, signaling the presence of damage. In response to bulky lesions, this stalling activates the TC-NER pathway. In the case of DSBs, dilncRNAs and DARTs play a crucial role in signaling and recruitment of repair factors such as BRCA1, BRCA2, and RAD51, facilitating resection of the broken DNA ends and strand invasion into the sister chromatid. The open chromatin state, transcription machinery, and the presence of these damage-induced RNAs also lead to the recruitment of chromatin re-modelers and histone modifiers, further enhancing the accessibility of the damaged site and promoting efficient repair [17]. Genome-wide, DNA damage induces the transcription of promoter-associated transcripts (PROMPTs) that facilitate gene repression [18]. Thus, active transcription, together with the synthesis of dilncRNAs and DARTs, not only identifies and signals the presence of DNA damage, but also creates a favorable environment for the assembly and function of DNA repair machinery. [14,19]. However, excessive transcription can lead to damage through cytosine de-methylation of exposed ssDNA, as well as DSBs, from abortive topoisomerase activity [20]. Pervasive transcription can also cause collisions with DNA replication machinery, leading to DNA-RNA hybrids (two stranded structures) and R-loops (three stranded structures composed of DNA-RNA hybrid and ssDNA), which further expose ssDNA to base deamination [21]. Therefore, the process of transcription in response to DNA damage must be tightly regulated. While transcription is crucial in the initial stages of repair, it must be reduced during repair and then restored again.

In this review, we discuss various mechanisms involved in transcription repression and its recovery in response to DNA damage.

## Transcriptional repression

Transcriptional repression following DNA damage has been extensively studied [22,23], revealing that temporarily shutting down transcription helps avoid collisions with DNA repair proteins and prevents the formation of dysfunctional transcripts from incomplete transcription of damaged genes [24,25]. The mechanisms employed by cells to repress transcription vary based on the extent and type of DNA damage.

### Transcriptional repression in response to transcription blocking lesions (TBLs)

UV irradiation can induce transcription-blocking lesions in DNA, such as 6–4 pyrimidine-pyrimidine (6-4PP) and cyclobutane pyrimidine dimers (CPDs) [26]. UV irradiation, as well as chemical agents like formaldehyde, can also form lesions between cross-linked proteins and DNA [27,28]. These lesions can halt the forward translocation of actively transcribing RNA Pol II via steric hindrance. As a result, elongating RNA Pol II (phosphorylated at serine 2 on the C-terminal domain of RNA Pol II or S2P RNA Pol II) stalls upon encountering these lesions. Transcription can only resume after the repair of these lesions through NER, specifically TC-NER. TC-NER triggers both a localized transcription repression at the damaged gene (*cis* effect) and a global transcriptional response (*trans* effect).

#### Transcriptional repression in cis to TBLs

TBLs on the template DNA strand represent roadblocks for RNA Pol II and must be resolved through TC-NER. Stalled RNA Pol II also obscures the DNA damage sites, preventing access of repair factors. When encountering these bulky lesions, the Cockayne syndrome complementation group B (CSB) protein attempts to propel RNA Pol II through the blockage via its 3’ to 5’ translocase activity. If CSB is unable to move RNA Pol II across the lesion, it associates stably with the elongating RNA Pol II, displacing elongation factor DSIF and switching from transcription elongation to TC-NER mode. Stalled RNA Pol II can either resume elongation after repair or undergo proteasomal degradation [29,30].

**Figure 1.**
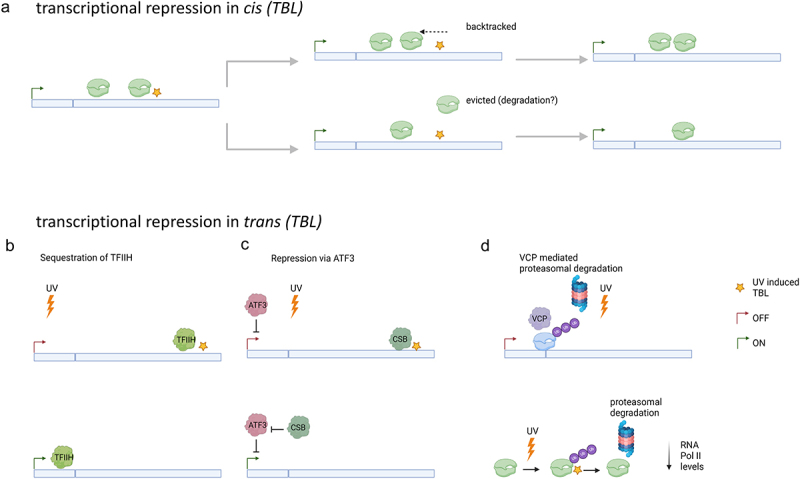
Mechanisms of transcriptional repression during UV-induced transcription blocking lesion (TBL).

Research using UV-damaged fibroblast cells indicates that stalled RNA Pol II is evicted from the template DNA strand during TC-NER [31]. Studies in XPC mutant cells, which can only repair TBLs through TC-NER, have shown that elongating RNA Pol II is displaced during repair. DRB treatment, which inhibits p-TEFb, was used to prevent the release of promoter-paused RNA Pol II (phosphorylated at serine 5 residue on the C-terminal domain of RNA Pol II or S5P RNA Pol II) [32]. The cells were treated with DRB for an extended period to allow the completion of transcription termination for all active elongating RNA Pol II molecules present on gene bodies. This was followed by a short incubation without DRB to enable the release of promoter-paused RNA Pol II. TBLs were then induced using UV irradiation. Elongating RNA Pol II densities across gene bodies at various time points after UV treatment were measured using mammalian native elongating transcript sequencing technology (mNET-Seq) [33]. If the stalled RNA Pol II were able to resume elongation following TC-NER, there would be a shift in RNA Pol II density from the 5’ end to the 3’ end of the gene over time, as all elongating RNA Pol II could reach transcription termination sites. However, only a non-significant increase in elongating RNA Pol II density near the 3’ end was observed, compared to a sharp decline at the transcription start site (TSS), indicating that RNA Pol II molecules were most likely evicted from DNA during TC-NER. Furthermore, results from excision repair sequencing (XR-seq), which indicate repair sites, [34], were consistent with the mNET-Seq data, showing no shift in the repair signal toward the 3’ end over time. These results suggest that RNA Pol II is evicted from the DNA strand, allowing RNA Pol II molecules queued behind to pass and encounter the next lesion, triggering TC-NER (Figure 1(a)).

a. RNA Pol II upon encountering a TBL can either backtrack or get evicted for potential degradation. Backtracked RNA Pol II may resume transcription following lesion repair. Created with Biorender.

b. Diminished transcription initiation due to sequestering of TFIIH at TBLs.

c. Transcription repression mediated by ATF3, which is upregulated upon UV damage. Sequestering of CSB at TBLs for TC-NER further allows repression through ATF3 as it cannot inhibit ATF3 present at gene promoters.

d. Reduced transcription initiation can result from VCP mediated proteasomal degradation of promoter bound RNA Pol II or due to depleted levels of RNA Pol II following proteasomal degradation of lesion stalled RNA Pol II.

In contrast to the RNA Pol II eviction theory, some studies [35,36] have reported that stalled RNA Pol II backtracks by 30–40 nucleotides and resumes transcription elongation after TBLs repair, a process mediated by the helicase activity of TFIIH. The backward translocase activity of TFIIH might conflict with the forward translocase activity of CSB, necessitating the removal of CSB from RNA Poll II. CSB can be removed during TC-NER through the ubiquitylation of its C-terminal domain, known as UBA [37]. Consistent with this hypothesis, mutants lacking the UBA domain showed incomplete recovery of RNA Pol II occupancy in the promoters of housekeeping genes compared to wild type cells. Thus, the inability to backtrack might impair TC-NER by preventing damage recognition by repair factors, leading to prolonged stalling of RNA Pol II. Persistent RNA Pol II stalling can result in its ubiquitylation and subsequent proteasomal degradation [38], reducing collisions with DNA replication machinery and limiting the accumulation of R-loops that endanger genome stability [39,40] (Figure 1(a)).

Although these mechanisms appear contradictory, both may be employed depending on the extend of DNA damage and the availability of repair factors like CSB and TFIIH. Severe UV damage may result in longer stretches of DNA lesions, such as consecutive pyrimidine dimers, prolonging the repair process to a point where RNA Pol II is selected for degradation or eviction. A similar situation may arise during extensive UV damage, leading to competition for repair factors, eventually resulting in prolonged stalling of RNA Pol II near unrepaired TBLSs Conversely, smaller lesions might allow backtracked RNA Pol II to resume transcription elongation without being degraded or evicted. The fate of RNA Pol II may thus depend on the time required for repair, influenced by factors such as lesion size and the availability of repair factors.

#### Transcriptional repression in trans to TBLs

UV-induced TBLs can also trigger a genome-wide response that modulates transcription initiation [41], splicing [42] and proximal promoter pausing [43]. This response in *trans* can be either TC-NER dependent or independent.

#### TC-NER dependent transcriptional repression in trans

One of the pioneering mechanistic studies on the global shutdown of transcription following UV damage was conducted using genome-wide Chromatin Immunoprecipitation sequencing (ChIP-Seq) analysis of RNA Pol II [41]. This study revealed a significant decline in RNA Pol II density around TSS approximately 2 h after UV irradiation. Subsequent studies confirmed the loss of promoter-bound S5P RNA Pol II around this time [44]. Unlike degradation, this loss was attributed to the eviction of TFIIH, which is essential for DNA strand opening during transcription initiation. The evicted TFIIH from promoters is then utilized in TC-NER to open the damaged strand and excise damaged bases. This was demonstrated by a reduced loss of promoter-bound S5P RNA Pol II when TC-NER proteins like CSA and CSB were knocked down, preventing TFIIH sequestration to TC-NER (Figure 1(b)). However, the study lacked comprehensive data supporting the absence of RNA Pol II degradation, showing only a reduction in S5P RNA Pol II levels by western blot analysis. Additionally, the study used a UV irradiation dose of 55 J/m^2^, which might not be ideal for studying *trans* effects since even a dose of 12 J/m^2^ can induce at least one TBL per gene, suggesting that observed phenomena might be due to *cis* effects of TBLs in promoters [44]. Interestingly, the study showed that p53-dependent DNA damage response genes did not show a loss in promoter-bound S5P RNA Pol II. The ability of p53 to activate transcription of its target genes in the absence of CTD kinases has been previously reported [45]. The transcription initiation of these genes was also unaffected by CSB depletion, indicating that p53 can regulate transcription independently of TC-NER [46]. Thus, transcription of DNA damage-responsive genes is safeguarded while housekeeping genes are shut down.

The reduction in transcription initiation of non-DDR genes could also be attributed to the UV-induced transcription repressor known as Activating transcription factor 3 (ATF3) [47,48]. ATF3 binds to CRE motifs in promoters and, by recruiting histone deacetylases, downregulates thousands of genes during UV irradiation [48]. ATF3 can be ubiquitylated in a CSB-dependent manner for proteasomal degradation. CSB sequesttration to DNA lesions, contributes to prolonged ATF3 mediated repression of transcription following UV damage [47] (Figure 1(c)).

Diminished transcription initiation following UV irradiation could also stem from the reduction in RNA Pol II levels due to degradation of lesion-stalled RNA Pol II [23,49]. Seminal work showed that degradation of stalled RNA Pol II is mediated through ubiquitilation of the K1268 residue of the RBP1 subunit of RNA Pol II [23,49]. Researchers modeled transcription upon UV irradation and compared their model to experimental data obtained by transient transcriptome sequencing (TT-seq). TT-seq is a variant of 4-thiouridine (4sU) sequencing. This approach labels newly synthesized RNA by metabolic incorporation of 4sU in live cells. Metagene profiles from TT-seq showed decreased nascent RNA production in promoter-paused regions approximately 3-h post-UV irradiation. Mutation of the K1268 residue almost fully restored the loss of promoter-bound RNA Pol II, demonstrating that degradation of RNA Pol II reduces the pool of RNA Pol II available for transcription initation. Cockayne Syndrome, characterized by an inability to restart transcription after UV damage due to mutations in the CSB gene [50], could result from the decline in RNA Pol II levels caused by continuous cycles of RNA Pol II stalling and degradation due to inefficient TC-NER. Indeed, introducing the K1268 mutation into CSB-deficient cells restored transcription recovery, further supporting this hypothesis. Interestingly, an increase in nascent RNA production in promoter-paused regions was observed along with a decline across the gene body 45 min post-UV induction, possibly indicating stalled RNA Pol II near lesions, or another *trans*-acting mechanism, negatively affecting the release of promoter-paused RNA Pol II. NELF-dependent upregulation of proximal promoter pausing has also been reported in response to DSBs, suggesting it might be beneficial to avoid further release of RNA Pol II, which would queue behind stalled RNA Pol II at TBLs.

#### TC-NER independent transcriptional repression in trans

Recent work [44] demonstrated genome-wide proteasomal degradation of promoter-paused RNA S5P Pol II through glycogen synthase kinase 3 (GSK3) signaling in response to UV damage. Using fluorescence recovery after photobleaching (FRAP) assays, the kinetics of GFP-tagged RNA Pol II upon UV damage were studied, discriminating between promoter-bound S5P RNA Pol II and elongating S2P RNA Pol II. The first hour post-UV damage showed a sharp reduction in S2P RNA Pol II mobilization, consistent with RNA Pol II stalling near lesions. After 1.5 h, the S5P RNA Pol II fraction also showed diminished mobilization. ChIP-Seq analysis further corroborated the loss of S5P RNA Pol II across all promoters, dependent on proteasomal degradation mediated by Valosin-containing protein (VCP). This degradation was restricted to the promoter-bound fraction, irrespective of whether the promoters had lesions (Figure 1(d)).

Additional studies [23] demonstrated that RNA Pol II degradation via ubiquitylation is the leading cause of transcription initiation loss, with VCP likely targeting the same ubiquitylated RNA Pol II residue for extraction and subsequent proteasomal degradation. Interestingly, this mechanism required productive transcription elongation for detecting lesions, but was not dependent on TC-NER factors like CSA and CSB. FRAP demonstrated the persistent immobilization of the S5P RNA Pol II fraction even after knockout of these TC-NER repair proteins, though this does not rule out an overall reduction in total RNA Pol II levels through degradation of persistently stalled RNA Pol II from defective TC-NER [23].

The degradation of lesion-stalled RNA Pol II, which could otherwise be evicted and reused for transcription initiation, might be responsible for the loss of the promoter-bound fraction. The delayed effect of genome-wide transcription repression compared to the *cis* effect allows most DNA damage response genes, which are short, to complete transcription within the initial 1.5 h window, while longer housekeeping genes are shut down. This observation suggests that DNA damage response genes may have evolve into a compact size to to be protected from transcriptional repression [23]. Compared to the indirect effect of genome-wide depletion of RNA Pol II, the GSK-mediated clearance of promoter-paused RNA Pol II remains constant across increasing DNA damage doses, evidenced by the comparable reduction in promoter-bound RNA Pol II fraction for both low and high UV doses. This suggests that the depletion of RNA Pol II through degradation of lesion-stalled RNA Pol II plays a secondary role in modulating transcription initiation upon UV damage in normal cells, becoming more prominent in TC-NER mutants where a greater fraction of lesion-stalled RNA Pol II is degraded.

### Transcriptional repression in response to double strand breaks

DSBs induced by irradiation, chemotherapy agents, and other stressors are repaired through ATM and PARP-mediated signaling processes. Unlike TBLs, caused by UV damage, DSBs trigger a distinct transcriptional repression program. The mechanisms activated upon sensing DSBs can be localized to a few hundred kilobases around the DSBs or can affect the genome more broadly.

#### Transcriptional repression in cis to DSBs

One of the first studies providing mechanistic insight into transcriptional repression near DSBs used a YFP-MS2 system to track nascent RNA synthesis after inducing cleavage of DNA with the *Fok*I endonuclease upstream of a reporter gene [22]. They observed a reduction in nascent RNA synthesis 3 h post-cleavage due to RNA Pol II stalling near the damaged reporter gene. ATM was shown to mediate repressive chromatin marks through monoubiquitylation of H2A at lysine 119 (H2AK119ub) in the area near DSBs, spanning multiple kilobases. Persistent ATM signaling was required to maintain this repressive chromatin environment, which would otherwise be deubiquitylated in the absence of ATM. The Polycomb Repressive Complex I was later identified as the downstream effector of the ATM signaling pathway, inducing H2AK119ub marks via its ubiquitin ligase components, RNF8 and RNF168 [51]. Further corroboration of ATM-mediated transcription repression upon DSB induction came from studies using the *Asi*SI model system, which allows for the induction of multiple sequence-specific DSBs [52,53]. In the *Asi*SI system, the restriction endonuclease *Asi*SI is fused to an estrogen receptor ligand-binding domain, which triggers its nuclear translocation upon addition of tamoxifen. This system enables precise and simultaneous cleavage of specific DNA sites upon tamoxifen treatment. Research utilizing this model system [52,53] demonstrated that transcriptional repression is inversely proportional to the distance from DSBs, affecting both transcription initiation and elongation, as shown by data from Cap Analysis of the Gene Expression sequencing (CAGE-Seq) [54] and Bromouridine Sequencing (Bru-Seq) [55] respectively. CAGE-Seq isolates and sequences transcripts containing 5’ cap structures for quantification of transcription initiation events, while Bru-Seq tracks nascent RNA production through incorporation of bromouridine into newly synthesized RNA. The propagation of transcriptional repression aligns with the theme of spreading repressive chromatin marks, as H2AK119ub can repress both initiation and elongation phases of transcription [56].

In contrast to studies reporting reductions in total RNA Pol II levels between damaged and undamaged cells, other studies did not observe such a reduction [22,52,57,58]. The absence of changes in RNA Pol II levels may be influenced by differences in repressive mechanisms based on the stages of repair. RNA Pol II degradation might be an immediate response, while ATM-mediated repressive chromatin marks establish a repressive environment during later stages of repair. Indeed, several studies [57–59] demonstrated rapid degradation RNA Pol II localized to the damaged gene harboring the DSB. Induction of DSBs at specific sites using the I-Ppol system showed a remarkable reduction in RNA Pol II occupancy (S2P RNA Pol II, S5P RNA Pol II, S7 RNA Pol II) in the damaged genes within half an hour of DSB induction [57–59]. The I-Ppol system uses the restriction enzyme I-Ppol fused to an estrogen receptor, allowing translocation to the nucleus to induce DSB at specific sites. The reduction in RNA Pol II levels within the damaged gene is orchestrated through DNA -dependent protein kinase (DNA-PK) mediated recruitment of the ubiquitin ligase WWP2 for the ubiquitylation and degradation of RNA Pol II via the proteasome. Interestingly, these studies did not observe a reduction in RNA Pol II occupancy in genes flanking the damaged gene, suggesting that this phenomenon is localized to DSB sites. Although the depletion of components required for the proteasomal degradation of RNA Pol II alleviated the rapid reduction in nascent RNA synthesis at the half-hour time point, transcriptional reduction near the DSBs was still observed 6 h post-damage. [57] This delayed repression may be mediated through ATM-dependent repressive chromatin marks [22]. A recent study further explored the dichotomy between the rapid localized degradation of RNA Pol II and the slower, more widespread effects of chromatin condensation [58]. In contrast to previous studies that used endonuclease systems to induce DSBs, this study employed a light-inducible CRISPR-Cas9 system to investigate transcription kinetics at a single specific DSB. Unlike the endonuclease-based system, which induces multiple DSBs in an unsynchronized manner, the CRISPR-Cas9 system is immediate and cleaves at only one site. The multiple DSBs introduced by the endonuclease system could mask the *cis* effect of a DSB through a global effect on transcriptional regulation, as reported in a previous study involving just 100 DSBs [60]. The kinetics study using MS2-GFP to measure the intensity of nascent RNA near the Cas9-induced DSB showed an exponential decay of transcription, leading to a complete loss of signal intensity within 5 min, along with the concomitant recruitment of the DNA repair protein G3BP1. This immediate transcriptional repression was not dependent on H2AK119ub, as knockdown of the histone ubiquitin ligases RNF8/RNF168 did not hinder this repression. Instead, it was dependent on the proteasomal degradation of RNA Pol II mediated through VCP. In contrast to UV damage, where VCP-mediated degradation of RNA Pol II, is important for the global shutdown of transcription [44], here it was necessary for the rapid shutdown of genes containing DSBs. ChIP analysis of RNA Pol II occupancy near the Cas9-induced DSB in the *ACTB* gene showed rapid propagation of the transcription repression signal at the rate 4.5 kb/min, eventually losing its potency around 500kb away from DSB. The propagation of the repression signal beyond *ACTB* was dependent on RNF8/RNF168-mediated repressive H2AK119ub marks, as evidenced by the lack of repression in the immediate flanking genes upon knockdown of these ubiquitin ligases. However, the repression of the DSB-harboring *ACTB*, which initiated in 2 min and plateaued at 30 min, did not depend on chromatin repressive marks. In fact, these marks were only induced until after 1 h.

Apart from ATM-mediated transcriptional repression, a few studies [61] have also detailed the role of Poly [ADP-ribose] polymerase 1 (PARP1) in establishing a transcriptionally repressive environment near the DSB. PARP1, an upstream regulator of DNA damage signaling [62], was shown to be required for recruitment of the negative elongation factor (NELF) to DSBs near transcriptionally active genes [61]. NELF can repress transcription elongation and keep RNA Pol II poised at the proximal promoter region [63]. The study also showed a reduction in poly ADP-ribosylation of NELF near the DSB, suggesting that the recruitment of PARP1 to DSBs alleviates poly ADP-ribosylation-dependent inactivation of NELF. Interestingly, the selective recruitment of NELF by PARP1 to DSBs on promoters of transcriptionally active genes hints that NELF is most likely recognizing the poly ADP-ribosylation marks on promoter bound RNA Pol II [64]. Hence, unlike the ATM-mediated spreading of repressive chromatin marks from the DSB, the NELF/PARP1-mediated repressive environment is more likely to be constrained only to the promoters of genes containing DSBs. Therefore, this mechanism likely works in synergy with ATM but provides an extra layer of transcriptional repression for genes with high transcriptional activity, such as housekeeping genes. Similar to ATM-mediated H2AK119ub modifications near the DSB, PARP1 can also induce repressive chromatin marks like H3K27Me3 [65]. PAR moieties on proteins deposited near DSBs can recruit CDYL1, which can mediate PRC2-dependent H3K27Me3 marks to condense chromatin near DSBs [65]. However, it remains to be seen whether these repressive marks can spread similarly to ATM-dependent H2AK119ub marks [22] or are constrained to regions with PAR moieties near DSBs.

**Figure 2.**
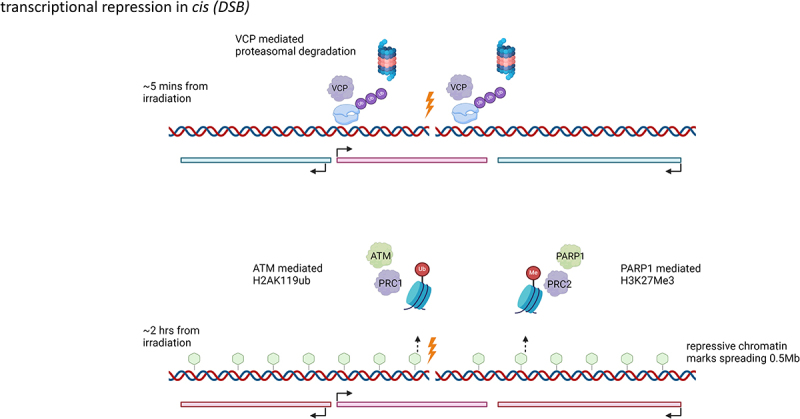
Mechanisms of transcription repression in *cis* after irradiation induced double strand breaks (DSB).

Overall, the transcriptional repression effect in *cis* near DSBs involves two layers of regulation: rapid proteasomal-mediated degradation of RNA Pol II localized to DSBs, and slower but broader effects utilizing chromatin modifications. The proteasomal degradation of RNA Pol II near the DSBs shares similarities with the degradation of persistently stalled RNA Pol II near TBLs induced by UV damage. However, the long-range spreading of repressive chromatin marks appears to be unique to transcriptional repression in *cis* near DSBs. These repressive chromatin marks would hamper TC-NER, as stalling of elongating RNA Pol II near TBLs is necessary for triggering the TC-NER signaling cascade. Hence, the transcriptional repression mechanisms might have evolved separately for different types of DNA damage (Figure 2).

VCP mediated proteasomal degradation of chromatin-bound RNA Pol II in the gene harboring DSB within 5 min of DSB induction. Establishment of repressive chromatin environment spanning 0.5Mb on either side of DSB after 2 s from DSB induction. Repressive chromatin environment can be in the form PARP1 mediated H3K27Me3 marks or ATM mediated H2AK119ub marks. Created with Biorender.

#### Transcriptional repression in trans to DSBs

**Figure 3.**
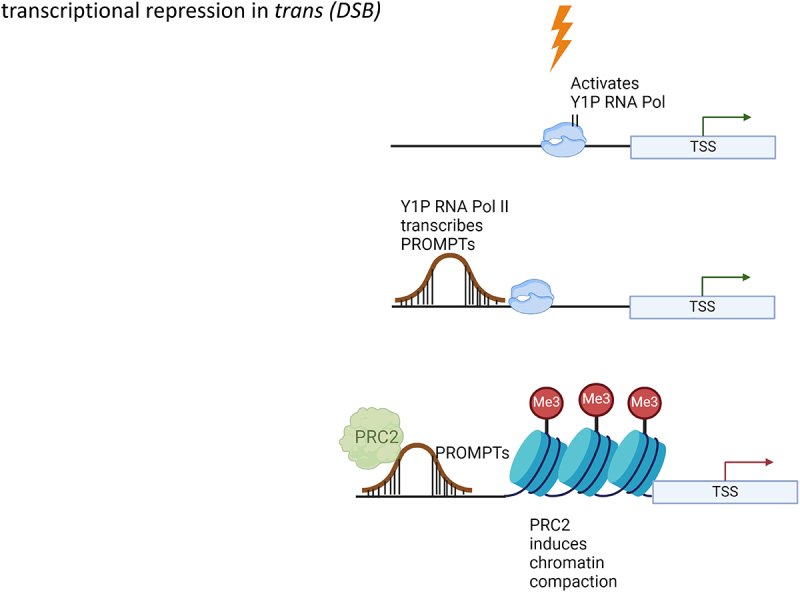
Mechanisms of transcription repression in *trans* after irradiation induced double strand breaks (DSB). Activated c-abl kinase triggers transcription of promoter associated antisense transcripts (PROMPTs) through Y1P RNA Pol II. PROMPTs recruit PRC2 to affect chromatin condensation and proximal promoter stalling of RNA pol II across the genome. Created with Biorender.

Although several studies have dissected transcriptional repression in *trans* during UV damage, few studies [18] have described this phenomenon during DSB induction. mNET-Seq analysis showed greater proximal promoter pausing of RNA Pol II, leading to significant downregulation of most protein-coding genes upon DSB induction using irradiation [18]. Interestingly, proximal promoter pausing is mediated through recruitment of PRC2 by promoter-associated antisense transcripts (PROMPTs), transcribed by tyrosine 1 phosphorylated RNA Pol II, which are upregulated in response to DSB induction (Figure 3). Elevated transcription near DSBs by Y1P RNA Pol II has been previously shown to be important for the recruitment of various DNA repair proteins [14]. This increased transcription has been attributed to the enhanced activity of c-ABL (the kinase responsible for phosphorylating the tyrosine 1 residue of RNA Pol II) through ATM-mediated signaling. It should be noted that irradiation might induce DSBs adjacent to promoters of protein-coding genes, and the observed effect might be influenced by other factors, including PARP1-mediated recruitment of NELF [61] and PRC2. Hence, future studies could involve restricting irradiation to a specific part of the nucleus using a microporous membrane, then examining the effects on promoter-paused RNA Pol II in the non-irradiated part. In line with UV damage studies, irradiation induced an upregulation of transcription of p53-dependent DNA damage response genes. Although these genes showed an increased promoter pausing index upon irradiation, there was also enhanced transcription initiation, as evidenced by the increased S5P RNA Pol II density near the promoter, leading to overall higher level of transcription elongation.

## Transcriptional re-activation

Transcription recovery after repression follows different kinetics and signaling mechanisms depending on the type of damage that induced the repression.

### Re-activation associated with the repair of transcription blocking lesions

Upon the induction of TBLs, the kinetics of transcriptional repression can be divided into two stages [44]. Initially, there is an immediate drop in nascent transcription due to lesion-stalled S2P RNA Pol II. This is followed by a further decrease due to the degradation of promoter-bound S5P RNA Pol II. Similarly, recovery is also divided into two phases. The recovery of S5P RNA Pol II begins around 4 h post-damage, with full transcription initiation restored by 6 h. However, the recovery of transcription elongation for lesion-stalled RNA Pol II begins later, around 6 h after damage induction, and takes approximately a day to complete [44].

**Figure 4.**
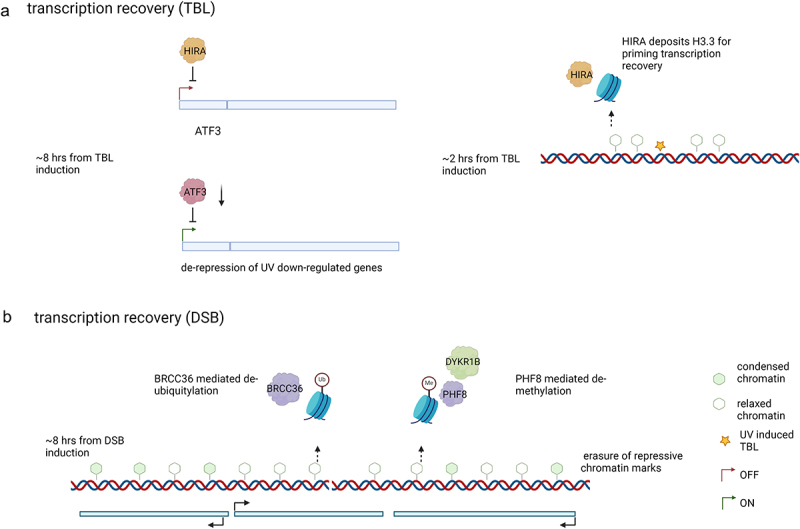
Mechanisms of transcription recovery following DNA damage.

As the repair of TLBs progresses, the various signaling processes that contribute to transcription repression diminish. Transcription repression decreases as the demand for TFIIHs for NER reduces. The freed-up CSB after repair could mediate the degradation of the transcription repressor ATF3 at promoters. Since the detection of TBLs by RNA Pol II is necessary for initiating the GSK-mediated degradation of promoter RNA Pol II, this process will also dissipate as repair progresses. In addition to the decay of signaling for transcription repression, cells have evolved active mechanisms to transition from transcription repression to recovery. The initial steps in transcription recovery begin as early as 30 min after damage induction, with the recruitment of histone regulator A (HIRA) to sites of DNA damage [66]. HIRA, a histone chaperone, facilitates the deposition of new H3.3 histone marks associated with active transcription [67]. Immunofluorescence assays using cells with localized UV damage showed accumulation of the HIRA complex at damage sites, along with a concomitant increase in H3.3 marks. The deposition of the HIRA complex at damaged sites was shown to depend on VCP, which mediated the degradation of UV-stalled RNA Pol II [68]. Recruitment of HIRA peaks about 1 h after damage induction and then decreases, while the newly established H3.3 marks remain. Sites with new H3.3 marks showed increased transcription about 20 h after damage, suggesting that H3.3 marks help prime damage sites for transcription recovery once repair is complete. However, the exact mechanisms through which H3.3 marks facilitate transcription recovery remain to be elucidated. Since H3.3 marks are associated with an open chromatin configuration [69], they might be deposited to allow access for repair factors rather than for transcription recovery. Interestingly, a follow-up study [68] on the role of the HIRA complex in transcription recovery demonstrated that the deposition of H3.3 is dispensable for transcription recovery post-UV damage. However, depletion of the HIRA complex still impaired the recovery of nascent transcription 20 h after UV damage induction. The authors showed that HIRA can transcriptionally repress ATF3, a transcriptional repressor inhibiting thousands of genes during UV damage. Inhibition of HIRA alleviated the repression of ATF3, which occurs in wild type cells 8 h after damage. HIRA inhibition did not affect transcription repression 2 h post-damage, further establishing that HIRA’s role in transcription recovery is independent of modulating transcription repression dynamics. Finally, the study concluded that the *trans* effect of HIRA is more important, as HIRA mutants without the ability to recognize damage sites did not delay transcription recovery. HIRA is also required for the recruitment of UBN2 (part of the HIRA complex), which deposits H3.3 marks at damaged chromatin. Although depletion of UBN2 disrupted transcription recovery, its role remains undefined since H3.3 marks at damaged sites are not necessary for recovery after damage. Even though knockdown of H3.3 did not affect nascent transcription levels genome-wide post UV damage, the localized effects on transcription at TBLs were not explored in prior studies. The role of H3.3 in transcription near damaged sites could be understood better using a site-specific inducible TBL model. Since VCP directs HIRA to damaged sites, it would be interesting to see if HIRA is also present at promoters as VCP can also target promoters during UV-induced DNA damage [44] (Figure 4(a)).

a. HIRA complex mediated transcription recovery following TBL induction. HIRA chaperones new H3.3 to damaged sites to prime the chromatin for later transcription recovery. HIRA can also repress ATF3, resulting in de-repression of UV downregulated genes, 8 h following TBL induction.

b. Repressive chromatin marks in form of H3K27Me3, H2AK119ub are erased through activity of PHF8 and BRCC36, respectively. The marks start to disappear around 8 h from DSB induction and coincide with fading of signaling mechanisms essential to maintain the repressive chromatin environment. Created with Biorender.

### Re-activation associated with double strand breaks repair

Similar to TBLs, the mechanisms of transcription recovery likely occur in phases. Repression of damaged genes through proteasomal degradation of RNA Pol II, mediated by various ubiquitin ligase [57,58], begins 5 min after damage. This rapid degradation of RNA Pol II was shown to plateau at 30 min and is restricted to the gene carrying the DSB. Although the plateauing of this repression mechanisms could coincide with the depletion of RNA Pol II near the break sites, the time of its eventual decline has not been reported yet. Since one of the studies [57] showed the dependency of this repression mechanism on DNA-PK signaling, it could depend on DSB repair.

After this rapid degradation, a repressive chromatin environment, extending several hundred kilobases from the DSB, mediated by RNF8/RNF168, ceases to spread further approximately 3 h after the DSB is induced. [22,58]. The RNF8/RNF168-mediated H2AK119ub chromatin marks lead to condensed chromatin and stalling of elongating RNA Pol II in genes neighboring the DSB. Persistent ATM signaling is needed to maintain this repressive environment, which would otherwise be resolved through the removal of repressive marks by the de-ubiquitylating enzyme BRCA1/BRCA2-Containing Complex Subunit 36 (BRCC36). Hence, this repressive mechanism should wane with the decline of ATM signaling as DSBs are repaired. A study [57] showed complete recovery of transcription of damaged and neighboring genes 6 h after DSB induction using I-Pol. However, more extensive DSB induction using irradiation could take up to 24 h to resolve the repressive chromatin environment.

Apart from the ATM-mediated repressive environment, PARP1 also induces a repressive chromatin environment in the form of H3K9me2 by recruiting the histone methyltransferase, Euchromatic histone-lysine N-methyltransferase 1 (EHMT) [70] to DSBs. A recent study [71] illustrates the recovery of transcription following DSB induction through the erasure of these marks. They showed the accumulation of H3K9me2 at laser micro-irradiated regions, which were abolished upon depletion of either EHMT or Dual specificity tyrosine-phosphorylation-regulated kinase 1B (DYKR1B), an upstream activator of EHMT. The onset of these marks is immediate, but interestingly, they also showed the simultaneous recruitment of the histone demethylase PHF8 to these damage sites in a DYKR1B-dependent manner. Depletion of PHF8, as expected, showed persistence of H3K9me2 at damaged sites, which are usually restored to non-damaged levels within 8 h. They also demonstrated delayed transcription recovery in genes adjacent to DSBs 8 h after DSB induction in PHF8-depleted cell using the *Asi*SI system. To separate the kinetics of transcription repression from transcription recovery, they incubated cells with tamoxifen for 3 h to induce DSBs and transcription repression. A 3 h incubation period with tamoxifen allows nuclear translocation of the *Asi*SI restriction enzyme conjugated with the estrogen nuclear receptor. The tamoxifen was then washed off to allow for repair and transcription recovery. Additionally, they did not observe a reduction in repair efficiency between wild type and PHF8-depleted cells, indicating that transcription recovery is not mediated through improved repair of DSBs. However, since both EHMT and PHF8 have antagonistic functions but are both recruited to DSBs by DYKR1B, additional regulatory mechanisms might be required to trigger the transition from transcriptional repression to recovery (Figure 4(B)).

## Conclusions

In this review, we compiled various studies on transcriptional responses during DNA damage. The transcriptional responses were categorized based on the type of DNA damage and whether it was localized near damage sites or genome-wide. Although various studies have illustrated different transcription repression mechanisms working in synergy during DSBs and TBLs repair, only a few have explored transcription recovery mechanisms. Particularly, in the case of UV damage, questions remain about how the evicted RNA Pol II near TBLs is repurposed for transcription initiation during recovery. Similarly, during DSB induction, future studies can investigate the fate of stalled elongating RNA Pol II on repressive chromatin marks and whether they can be recycled for transcription initiation through changes in CTD phosphorylation marks. The signaling mechanism for the transition from repression to recovery can also be explored, especially in the case of DSBs. More importantly, how does the cell maintain a genome-wide “memory” of the unperturbed transcription state to return to following damage-induced repression? Another major facet of transcription during the DDR is the nature of non-coding transcripts generated from DSBs (DARTs) [72] and how they are temporally regulated with respect to transcription repression. Future studies utilizing a light inducible CRISPR-Cas9 system with multiple DSB sites [60] could provide the temporal and spatial resolution needed to address such questions.
